# Estimation of phenotypic variability in symbiotic nitrogen fixation ability of common bean under drought stress using ^15^N natural abundance in grain

**DOI:** 10.1016/j.eja.2016.05.014

**Published:** 2016-09

**Authors:** Jose Polania, Charlotte Poschenrieder, Idupulapati Rao, Stephen Beebe

**Affiliations:** a Centro Internacional de Agricultura Tropical (CIAT), A. A. 6713 Cali, Colombia; b Universidad Autónoma de Barcelona, Lab Fisiología Vegetal, Facultad de Biociencias, Bellaterra, Spain

**Keywords:** Shoot biomass, Nitrogen derived from the atmosphere, Nitrogen derived from the soil, Nitrogen use efficiency, Terminal drought stress

## Abstract

Common bean (*Phaseolus vulgaris* L.) is the most important food legume, cultivated by small farmers and is usually exposed to unfavorable conditions with minimum use of inputs. Drought and low soil fertility, especially phosphorus and nitrogen (N) deficiencies, are major limitations to bean yield in smallholder systems. Beans can derive part of their required N from the atmosphere through symbiotic nitrogen fixation (SNF). Drought stress severely limits SNF ability of plants. The main objectives of this study were to: (i) test and validate the use of ^15^N natural abundance in grain to quantify phenotypic differences in SNF ability for its implementation in breeding programs of common bean with bush growth habit aiming to improve SNF, and (ii) quantify phenotypic differences in SNF under drought to identify superior genotypes that could serve as parents. Field studies were conducted at CIAT-Palmira, Colombia using a set of 36 bean genotypes belonging to the Middle American gene pool for evaluation in two seasons with two levels of water supply (irrigated and drought stress). We used ^15^N natural abundance method to compare SNF ability estimated from shoot tissue sampled at mid-pod filling growth stage vs. grain tissue sampled at harvest. Our results showed positive and significant correlation between nitrogen derived from the atmosphere (%Ndfa) estimated using shoot tissue at mid-pod filling and %Ndfa estimated using grain tissue at harvest. Both methods showed phenotypic variability in SNF ability under both drought and irrigated conditions and a significant reduction in SNF ability was observed under drought stress. We suggest that the method of estimating Ndfa using grain tissue (Ndfa-G) could be applied in bean breeding programs to improve SNF ability. Using this method of Ndfa-G, we identified four bean lines (RCB 593, SEA 15, NCB 226 and BFS 29) that combine greater SNF ability with greater grain yield under drought stress and these could serve as potential parents to further improve SNF ability of common bean.

## Highlights

• New method was used to estimate symbiotic nitrogen fixation ability in bean.
• Phenotypic variation in nitrogen fixation was observed under drought stress.
• Four lines were superior in their ability to fix nitrogen under drought stress.
• Several lines were found to combine drought resistance with ability to fix nitrogen.

## 1 Introduction

Common bean (*Phaseolus vulgaris* L.) is the most important food legume cultivated in the tropics of Latin America and eastern and southern Africa. It is nutritionally rich in iron and protein, and is a source of fiber and carbohydrates that are essential in the nutrition of the population, especially in developing countries. The bean crop is cultivated by small farmers and it is often exposed to unfavorable conditions with minimum use of inputs (Beebe et al., 2013, Beebe et al., 2008). Bean yields are affected by various biotic and abiotic stress factors. Drought affects about 60% of the bean producing regions causing yield losses between 10 and 100%. Drought is the second most important factor in yield reduction after disease (Rao, 2014, Thung and Rao, 1999). In addition to drought, smallholder bean production is often affected by low soil fertility in marginal areas (Douxchamps et al., 2010) where the majority of grain legumes are cultivated (Beebe et al., 2014, Sinclair and Vadez, 2012). About 50% of the bean production areas in Eastern and Central Africa and 60% in Southern Africa are subjected to N deficiency due to both depletion of N in the soil and application of limited N fertilizer (Beebe et al., 2014). Thus the ability of the crop to acquire N from the soil is limited. Common bean can supply at least part of its N requirement through symbiotic nitrogen fixation (SNF). However compared to other legumes, beans have lower SNF capacity (Hardarson, 2004, Hardarson et al., 1993, Peoples et al., 2009). The estimated mean value of nitrogen derived from the atmosphere (Ndfa) for common bean across different geographical regions of the world is 39% (Peoples et al., 2009). This value is in contrast with the Ndfa values of 54%–65% observed for other widely-grown legume crops and to the values recorded for soybean and faba bean which were 68% and 75%, respectively (Peoples et al., 2009).

Abiotic and biotic stress factors such as P deficiency, drought, and pest and diseases affect SNF capacity (Ramaekers et al., 2013). Among these limitations, SNF is highly sensitive to drought (Beebe et al., 2014, Devi et al., 2013), with possible interactions among stresses. Bean genotypes with resistance to drought are affected by N deficiency, and SNF capacity is affected by drought stress (Devi et al., 2013). Moreover, drought has a negative influence on both the rhizobia and the nodulation process of legumes (Sinclair and Vadez, 2012). Drought even can cause the loss of SNF activity in common bean, and other legume species that generally have low rates of N fixation even under well-watered conditions (Devi et al., 2013). Identification of parental genotypes to use in breeding that combine superior SNF ability under drought stress with other desirable traits could be a useful strategy to confront the new challenges of climate variability and to ensure food security in marginal areas.

Different techniques have been used to estimate SNF, including N_2_ balance, N_2_ difference, ^15^N natural abundance, ^15^N isotope dilution, ureide analysis, acetylene reduction assay and hydrogen evolution (Unkovich et al., 2008). The ^15^N natural abundance method presents some advantages over other methodologies. It can be applied under both greenhouse and field conditions, allows estimation of N fixation in almost any situation where both N-fixing and non N-fixing plants are present at the same location, and it can be applied to farmers’ fields, or to any field experiments where legumes and non N-fixing plants coexist (Douxchamps et al., 2010, Unkovich et al., 2008). Also, the ^15^N natural abundance method allows to separate N derived from the atmosphere with the N derived from soil (Boddey et al., 2000). To calculate the total contribution of SNF in kg ha^−1^, the estimation of shoot biomass in kg ha^−1^ is required (Boddey et al., 2000, Unkovich et al., 2008). This methodology is usually applied to shoot tissue of the plant harvested at different growth stages such as flowering or pod filling (Boddey et al., 2000, Unkovich et al., 2008). Generally shoot tissue is used because to use the whole plant including roots is complex. However, the fact of taking shoot biomass sample, from a breeding perspective, is equally complex because of the large sample size for destructive sampling of the plot. This could mean significant labor costs for plant breeding programs dealing with large numbers of breeding lines. For these reasons most bean breeding programs do not routinely select for better SNF ability. Developing methodologies that can estimate SNF ability using grain tissue could be easier to integrate into most breeding programs since grain is routinely harvested to estimate yield and/or nutritional quality. Therefore based on the principle that common beans mobilize much of their N from vegetative structures to the grain (Lynch and White, 1992, Ramaekers et al., 2013), we propose that it would be much easier to apply the methodology of ^15^N natural abundance using the grain tissue at the time of harvest.

The main objectives of this study were to: (i) test and validate the use of ^15^N natural abundance in grain to quantify phenotypic differences in symbiotic nitrogen fixation (SNF) ability for its implementation in breeding programs aiming to improve SNF in common bean, and (ii) quantify phenotypic differences in SNF under drought stress to identify superior genotypes that could serve as parents.

## 2 Materials and methods

### 2.1 Experimental site and meteorological conditions

Two field trials were conducted during the dry season from June to September in two years (2012 and 2013), at the main experimental station of the International Center for Tropical Agriculture (CIAT) in Palmira, Colombia, located at 3° 29ʺ N latitude, 76° 21ʺ W longitude and an altitude of 965 m. Basic characteristics of this field site were described previously (Beebe et al., 2008). The soil is a Mollisol (Aquic Hapludoll) with 23.3 g kg^−1^ of organic matter and no limitations of availability of iron (Fe) and molybdenum (Mo) for the process of SNF. Common bean has been grown on this field for many years and there is adequate native *Rhizobium* in soil with 5.2 × 10^6^ colony forming units of rhizobia per gram of soil. During the crop-growing season, maximum and minimum air temperatures in 2012 were 31.0 °C and 19.0 °C, and in 2013 were 30.2 °C and 19.2 °C, respectively. Total rainfall during the active crop growth was 85.8 mm in 2012 and 87.7 mm in 2013. The potential pan evaporation was of 385.2 mm in 2012 and 351.0 mm in 2013. Two levels of water supply (irrigated and drought) were applied through furrow irrigation (approximately 35 mm of water per irrigation). The drought stress treatment in 2012 received 3 irrigations (at 3 days before sowing and at 5 and 23 days after sowing). In 2013, irrigation was provided at 3 days before sowing and at 4 and 15 days after sowing. In both years, irrigation was suspended after the application of the third irrigation to induce terminal drought stress conditions (less water availability from flowering to physiological maturity). The irrigated control treatment received 5 irrigations in 2012 and 6 irrigations in 2013 to ensure adequate soil moisture for crop growth and development.

### 2.2 Plant material and experimental design

For this study 36 bush bean genotypes belonging to the Middle American gene pool were selected: twenty two elite lines of common bean (BFS 10, BFS 29, BFS 32, BFS 67, MIB 778, NCB 226, NCB 280, RCB 273, RCB 593, SCR 16, SCR 2, SCR 9, SEN 56, SER 118, SER 119, SER 125, SER 16, SER 48, SER 78, SMC 141, SMC 43 and SXB 412); five interspecific lines from the cross between elite line SER 16 and *Phaseolus coccineus* (ALB 6, ALB 60, ALB 74, ALB 88 and ALB 213); one landrace of tepary bean (*Phaseolus acutifolius*) G 40001 from Veracruz-Mexico, and two interspecific lines between tepary bean and common bean (INB 841 and INB 827 developed from five cycles of congruity backcrossing of tepary with ICA Pijao). BFS (small red) lines were developed to improve adaptation to low soil fertility and drought. SER and SCR (small red), SEN (small black) and NCB (small black) lines were developed for improved adaptation to drought. ALB (small red) lines were developed for improved adaptation to drought and aluminum toxicity. RCB (small red) lines were developed for improved yield potential, disease resistance and commercial grain. SEA 15 and BAT 477 were included as drought resistant checks, and three commercial cultivars of common bean (DOR 390, Pérola and Tio Canela) as drought sensitive materials. BAT 477 NN was included as a non-fixing bean genotype which was used as reference plant to estimate nitrogen derived from the atmosphere (Ndfa). In the two years, a 6 × 6 partially balanced lattice design with 3 replications was used. Experimental units consisted of 4 rows with 3.72 m row length with a row-to-row distance of 0.6 m and plant-to-plant spacing of 7 cm (equivalent to 24 plants m^−2^). Trials were weeded and sprayed with insecticides and fungicides as needed.

### 2.3 Determination of symbiotic nitrogen fixation ability using shoot and grain

To compare and validate the method of ^15^N natural abundance, we sampled shoot tissue at mid-pod filling and grain tissue at harvest time. We sampled a representative plant within a row of 50 cm long at mid-pod filling and also at harvest time for each genotype and from each plot of both irrigated and drought treatments for oven drying and grinding and for ^15^N estimation. The plant was cut at the soil surface, washed with deionized water and dried in the oven at 60 °C for two days. The dried sample was finely ground using a ball-mill and was weighed using a microbalance to pack 2.5 mg of each sample in a tin capsule. These samples in tin capsules were sent to UC Davis Stable Isotope Facility in USA for ^15^N isotope analyses. The percentage of N derived from the atmosphere (%Ndfa) was determined for both shoot and grain samples using the ^15^N natural abundance method (Shearer and Kohl, 1986). BAT 477 NN was used as a non-fixing reference plant.

$$\%\text{Ndfa}=\frac{\delta \text{N}\text{non fixing reference plant}\;-\beta }{\delta^{15}}\times 100$$

Where β is the δ^15^N value from the nitrogen fixing bean plant grown in N free medium. The isotope discrimination occurs internally within the plant so that the different plant parts differ in δ^15^N (Unkovich et al., 1994). Consequently, different β values were used to estimate %Ndfa for the shoot at mid-pod filling and grain at harvest. The β values used were −3.09‰ for shoot at mid-pod filling and −2.44‰ for grain at harvest for genotypes with growth habit II and −3.62‰ for shoot at mid-pod filling and −2.88‰ for grain for genotypes with growth habit III. The β values were generated from conducting a pot experiment in the greenhouse at CIAT, following the procedure of Unkovich et al. (1994). We used SMC 140 and GGR 18 as representative genotypes of growth habit II and III, respectively. Total shoot and seed N content per unit area (kg ha^−1^) were estimated using the values of N concentration in shoot biomass and grain and dry weights of shoot biomass and grain. Total N derived from atmosphere in kg ha^−1^ (TNdfa) and total N derived from soil in kg a^−1^ (TNdfs) were estimated (Unkovich et al., 1994). Nitrogen use efficiency (NUE) was estimated as kg of grain produced per kg of shoot N uptake at mid-pod filling growth stage.

### 2.4 Shoot biomass and grain yield measurements

At mid-pod filling, a 50 cm segment of the row from each plot with about 7 plants was used for destructive sampling to measure shoot biomass (SB). Also at mid-pod filling, the roots of three plants per plot (selected randomly) of the non-fixing bean genotype (BAT 477 NN) were pulled from soil to check for the absence of nodules. At the time of harvest, plants in 50 cm of a row from each plot were cut and dry weights of stem, pod, seed, and pod wall were recorded. Grain was harvested from two central rows after discarding end plants in both the irrigated and drought plots. In order to compare shoot dry biomass with grain dry weight, mean values of grain yield per hectare were corrected for 0% moisture in grain.

### 2.5 Statistical analysis

All data were analyzed using the SAS (v 9.0) PROC MIXED and PROC CORR (SAS Institute Inc., 2008). The adjusted means for each genotype and the environment (irrigated and drought) were obtained using the mixed models theory together with the MIXED procedure considering the effects of the replications and blocks within replications as random and genotypes as fixed. Correlation coefficients were calculated by the PROC CORR. In the following text, values marked with *, ** or *** are statistically significant at probability levels of 5%, 1% and 0.1%, respectively.

## 3 Results

### 3.1 Estimation of Ndfa and differences in ^15^natural abundance in shoot and grain

Analysis of %N derived from the atmosphere (%Ndfa) estimates the proportional dependence of the biomass N on N_2_ fixation. The %Ndfa in grain was compared with %Ndfa in shoot biomass to determine their relationship. If the %Ndfa in grain is closely related with %Ndfa in shoot, legume breeders would be able to select for SNF based on the grain values without the need to harvest, dry and grind large volumes of shoot biomass. A significant and positive correlation values of r = 0.81*** in 2012 and r = 0.66*** in 2013 (r = 0.83*** for combined data for two seasons, Table 1) were observed between the %Ndfa values estimated with ^15^N natural abundance of shoot biomass at mid-pod filling growth stage and %Ndfa values estimated with ^15^N natural abundance in the grain under irrigated conditions; the correlation values were also significant and positive under drought conditions r = 0.67*** in 2012 and r = 0.74*** in 2013 (r = 0.71*** for combined data for two seasons, Table 1).

Nodule formation was observed in both irrigated and drought treatments in both years and with all the genotypes evaluated, except for BAT477 NN which was used as a non-nodulating reference plant for estimating SNF ability. Significant differences were observed in both shoot and grain δ^15^N between the non-fixing bean genotype and the other lines tested under both irrigated and drought conditions (Table 2). The values of δ^15^N for shoot for the non-fixing bean genotype (BAT 477_NN) in 2012 were 5.6 and 8.7 under irrigated and drought conditions, respectively (Table 2); and in 2013 the values were 8.3 and 9.5 under irrigated and drought conditions, respectively (Table 2). Under irrigated conditions, the δ^15^N for shoot of 35 genotypes (Excluding BAT 477_NN) ranged from −0.2 to 1.7 in 2012 and from 2.1 to 5.6 in 2013 (Table 2). Under drought conditions the δ^15^N for shoot of 35 genotypes ranged from 3.6 to 7.5 in 2012 and from 4.3 to 8.5 in 2013 (Table 2). The values of δ^15^N for grain for BAT 477_NN in 2012 were 5.8 and 8.6 under irrigated and drought conditions, respectively (Table 2); and in 2013 the values were 6.8 and 8.3 under irrigated and drought conditions, respectively (Table 2). Under irrigated conditions, the δ^15^N for grain of 35 genotypes ranged from 0.0 to 1.4 in 2012 and from 1.7 to 4.8 in 2013 (Table 2). Under drought conditions, the δ^15^N for grain of 35 genotypes ranged from 4.5 to 8.0 in 2012 and from 4.4 to 7.0 in 2013 (Table 2).

### 3.2 Differences in SNF ability and genotypic response to drought

**Fig. 1:**
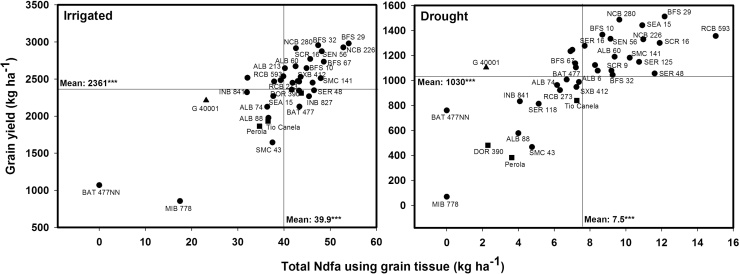
Identification of genotypes that combine greater total nitrogen derived from the atmosphere in kg ha^−1^ estimated using grain tissue (TNdfa-G) with superior grain yield under irrigated and drought conditions when grown in a Mollisol at CIAT-Palmira, Colombia. Higher TNdfa-G genotypes with greater grain yield were identified in the upper, right hand quadrant. Genotypes identified with symbols of (■) are commercial varieties and with a symbol of (▲) is *P. acutifolius.*

**Fig. 2:**
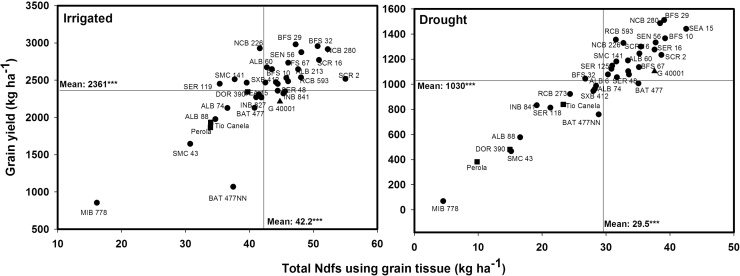
Identification of genotypes that combine greater total nitrogen derived from the soil in kg ha^−1^ estimated using grain tissue (TNdfs-G) with superior grain yield under irrigated and drought conditions when grown in a Mollisol at CIAT-Palmira, Colombia. Higher TNdfs-G genotypes with greater grain yield were identified in the upper, right hand quadrant. Genotypes identified with symbols of (■) are commercial varieties and with a symbol of (▲) is *P. acutifolius.*

**Fig. 3:**
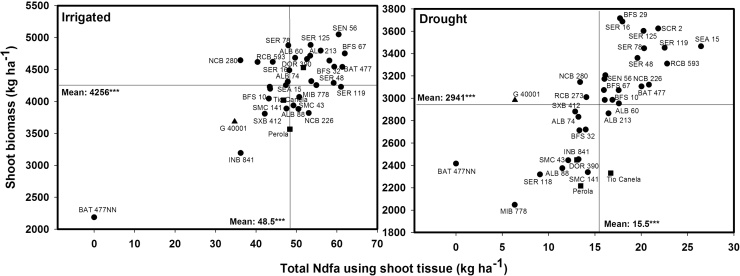
Identification of genotypes that combine greater total nitrogen derived from the atmosphere in kg ha^−1^ estimated using shoot tissue (TNdfa-SH) with superior grain yield under irrigated and drought conditions when grown in a Mollisol at CIAT-Palmira, Colombia. Higher TNdfa-SH genotypes with greater grain yield were identified in the upper, right hand quadrant. Genotypes identified with symbols of (■) are commercial varieties and with a symbol of (▲) is *P. acutifolius.*

An average reduction of 70% in 2012 and 38% in 2013 in SNF ability was observed under drought stress in bush Middle American genotypes using the grain method (Table 2). The lines RCB 593, BFS 32, SER 125, SMC 141 and BFS 29 maintained a relatively higher level of SNF ability under drought stress in both years (Table 2). A weak correlation was observed between %Ndfa estimates using grain samples and grain yield under irrigated conditions (Table 1). No correlation was observed between %Ndfa estimates and grain yield under drought conditions (Table 1). However, the lines RCB 593, SEA 15, NCB 226 and BFS 29 were superior in combining high values of grain yield with greater values of %Ndfa under drought stress. The line NCB 226 was superior in %Ndfa ability under both irrigated and drought conditions (Table 2). The accumulation of N (kg ha^−1^) in grain was reduced by 55% due to drought stress in Middle American bush beans, being more sensitive the accumulation of total N in grain derived from the atmosphere (TNdfa-G) than N in grain derived from soil (TNdfs-G), with reduction of 78% and 43%, respectively (Fig. 1, Fig. 2). Under irrigated conditions the lines BFS 29, SCR 16, BFS 32, NCB 280 and SEN 56 presented higher total N content in grain from both TNdfs and TNdfa values compared with the other lines tested (Fig. 1, Fig. 2). *Phaseolus acutifolius* (G 40001) was outstanding in its ability for TNdfs, and showed a drastic decrease in TNdfa under drought (Fig. 1, Fig. 2). The lines SEA 15, RCB 593 and BFS 10 maintained higher total N content in grain for both TNdfs and TNdfa values, compared with the other lines tested under drought stress (Fig. 1, Fig. 2). The three commercial varieties (DOR 390, Tio Canela 75 and Perola) presented lower N content in grain for both TNdfs and TNdfa values under both irrigated and drought conditions (Fig. 1, Fig. 2). Similar tendency was observed in the relationship between shoot biomass and total Ndfa estimated using shoot tissue (TNdfa-SH) (Fig. 3). Genotypes that stood out for a higher TNdfa-G and grain yield under both irrigated and drought conditions (Fig. 1, Fig. 2) also exhibited higher TNdfa-SH and shoot biomass at mid-pod filling under both irrigated and drought conditions (Fig. 3). Several inbred lines were superior in their shoot biomass, grain yield and TNdfa-SH and TNdfa-G than the three commercial varieties (DOR 390, Tio Canela and Perola) under both irrigated and drought conditions (Fig. 1, Fig. 2, Fig. 3).

Several genotypes including BFS 29, SEN 56, NCB 226, NCB 280, SCR 16, ALB 60, RCB 593 and SER 48 presented higher total N content in grain under irrigated conditions, and also maintained N levels well under drought conditions. The lines MIB 778, Pérola, SMC 43, ALB 88, Tio Canela and the non-nodulant BAT477NN showed low grain N content under both irrigated and drought conditions. The lines SEA 15, NCB 280, BFS 10, SEN 56, BFS 29, NCB 226 and SER 16 not only showed high values in grain for TNdfs but also were outstanding in their TNdfa value using grain that resulted in greater values of grain yield under drought stress, while RCB 593 combined higher values of TNdfa and an intermediate level of Ndfs with greater grain yield under drought conditions (Fig. 1, Fig. 2).

**Fig. 4:**
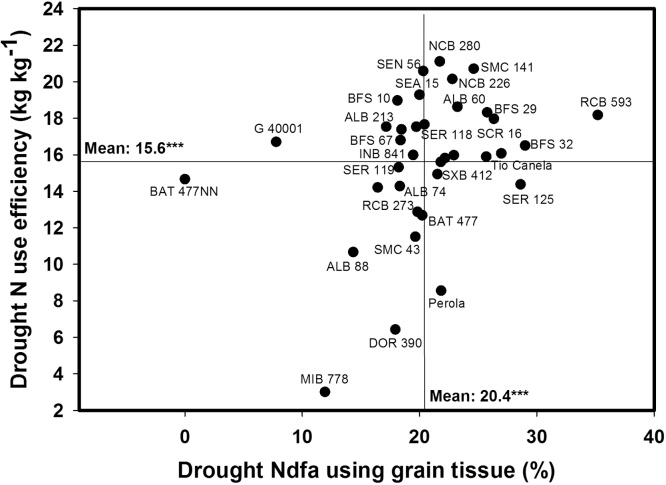
Identification of genotypes that combine greater values of %nitrogen derived from the atmosphere using grain tissue (%Ndfa-G) with higher values of nitrogen use efficiency (NUE) in terms of kg of grain produced kg^−1^ of shoot N uptake under drought conditions when grown in a Mollisol at CIAT-Palmira, Colombia. Higher%Ndfa-G genotypes with greater values of NUE were identified in the upper, right hand quadrant.

A positive and highly significant correlation was observed between grain yield and nitrogen use efficiency (NUE) under both irrigated and drought conditions (Table 1). Genotypes that combined better grain yield with higher NUE under drought conditions were RCB 593, SMC 141, BFS 32, BFS 29, SEA 15, SEN 56, NCB 280 and NCB 226. A low but significant correlation coefficient (r = 0.19**) was observed between %Ndfa and NUE under drought conditions. The lines RCB 593, SMC 141, BFS 32, BFS 29, SEA 15, SEN 56, NCB 280 and NCB 226 combined higher values of NUE and %Ndfa under drought stress (Fig. 4). The commercial cultivar Pérola showed a moderate value of %Ndfa but it was poor in its NUE and was low yielding under drought (Fig. 4).

## 4 Discussion

This study allowed to compare the estimation of %Ndfa using shoot tissue (%Ndfa-SH) vs. grain tissue (%Ndfa-G), to quantify phenotypic differences in common bean for SNF ability under irrigated and drought stress conditions, and to test whether %Ndfa-G could be a useful trait in breeding programs. Results from two seasons with %Ndfa values estimated under field conditions, comparing the conventional methodology based on shoot tissue at the growth stage of mid-pod filling (%Ndfa-SH), and the method proposed here using grain tissue at the time of harvest (%Ndfa-G), showed that the latter methodology is feasible. The high correlation between both methods validates this statement. We suggest that grain samples collected at harvest can be used to quantify phenotypic differences in SNF ability in common bean using the methodology of natural abundance of ^15^N. The proposed %Ndfa-G method, from a breeding perspective, is much easier than the conventional methodology involving shoot tissue (%Ndfa-SH) which requires destructive sampling, drying of fresh tissue, sampling of plant parts for grinding, all with more labor and therefore is less cost effective. With the proposed %Ndfa-G methodology, the breeder can take a sample of harvested grain, dry and grind it and analyze for the isotope ratios of δ^13^C and δ^15^N, simultaneously selecting for water use efficiency (Araus et al., 2002, Easlon et al., 2014) and SNF ability (based on higher values of %Ndfa). In a recent genomic study addressing SNF ability in common bean, Kamfwa et al. (2015) using both shoot and grain samples suggested selection for high SNF ability based on grain tissue could be easier integrated into most breeding programs. Similar results for %Ndfa using the ^15^N natural abundance technique in grains and the whole shoots have also been reported for other legume species (Bergersen et al., 1985). Discordant results reported by others can occur when grain filling during the late stage of development is highly dependent on the contribution of N fixation rather than on the extent of remobilization of N from vegetative structures (Bergersen et al., 1992).

Previous research showed that common beans are able to translocate about 80–93% of its total N to the grain (Ramaekers et al., 2013). Also it has been shown that common bean accumulates preferentially the fixed N into the grain (Dubois and Burris, 1986, Westermann et al., 1985, Wolyn et al., 1991). At such high rates of N translocation in common beans, trends in %Ndfa would be similar between shoot tissue and grain tissue. Several of the lines evaluated in this study, have high mobilization of N from shoot to grain under both irrigated and drought conditions, as can be evidenced from our results, where the genotypes with greater N accumulation in shoot at mid-pod filling also showed greater values of N accumulation in grain at harvest. The contrary was true with the genotypes that accumulated less N in shoot and grain.

This study also permitted evaluating the SNF ability in a set of elite common bean breeding lines that were recently developed for improving resistance to drought. Furthermore, we tested lines that were derived from crosses among bean races (Beebe et al., 2008), as well as interspecific crosses with introgression from *P. coccineus* (Butare et al., 2012) or *P. acutifolius* (Beebe, 2012). Several lines developed over different breeding cycles to improve drought resistance not only performed better under water shortage, but also had higher ability to fix N under these conditions. Under drought stress,both grain yield and total Ndfa in these lines doubled the values that were observed for three leading commercial cultivars grown in Latin America: DOR 390, Perola, and Tío Canela. Under unfavorable conditions, such as drought, a decrease in the effectiveness of SNF process is expected (Devi et al., 2013). The symbiosis is based on the carbon supply from the plant to the Rhizobium which provides fixed N to the plant. But under drought stress, the reduced net photosynthesis decreases the supply of photosynthates to the nodules resulting in lower values of %Ndfa (Gonzalez et al., 1995, Sassi et al., 2008). Our results confirm previous reports that SNF in common bean is especially sensitive to drought stress, as drought reduced Ndfa by 57%. SNF is a physiological process especially sensitive to soil drying (Devi et al., 2013), and the effect of drought stress on SNF varies according to the stages of development when the stress occurs. Water stress during early vegetative growth had no significant effect on SNF, but during flowering or grain filling it significantly reduced fixed N (Chalk et al., 2010). In the two growing seasons of this study the drought stress was imposed starting at the critical preflowering stage, resulting in high inhibition of SNF ability.

The superior performance of a few drought-adapted lines that combine greater grain yield with higher values of %Ndfa in grain might be due to greater carbon transport to both grain and the nodule. It is also noteworthy that the drought resistant bean lines with higher SNF ability had greater values of shoot biomass indicating the importance of plant vigor for supporting nodule development, as well as contributing to remobilization of both C and N to developing grains. Vigorous plants permit higher levels of N accumulation of both fixed N and non-fixed N, while maintaining or improving remobilization of this N to grain, contributing to increased grain yield in common bean (Wolyn et al., 1991). Higher shoot biomass and N accumulation before pod set could provide an advantage for drought adapted lines (Vadez et al., 2014). The lower sensitivity of SNF ability in drought resistant lines can be explained by the expeditious removal of N products from nodules and sequestering them in the shoot of the plant to avoid N-feedback limitation on SNF (Beebe et al., 2014, Sinclair and Vadez, 2012).

Tepary bean (*P. acutifolius*) has been reported to present multiple traits for drought resistance including early maturity, greater photosynthate remobilization capacity, deep rooting, small leaves, and stomatal control for improved water use efficiency (Beebe et al., 2014, Mohamed et al., 2005, Rao et al., 2013). But in spite of excellent drought resistance, our results indicate that *P. acutifolius* drastically reduces its SNF under drought, suggesting an internal control of SNF, possibly through decreased carbon supply to the nodules, while maintaining uptake of mineral N from soil through its fine root system. In early maturing genotypes, competition for photosynthates exists between nodules and developing pods/grain (Piha and Munns, 1987, Vadez et al., 2014). *Phaseolus acutifolius* is early maturing, and may preferentially channel the photosynthates into pods and grains rather than destine them to the development and maintenance of the nodules. This in part could be an evolutionary response to the hot and dry environments from where tepary bean originated.

Common beans are poor N fixers compared to other grain legumes (Hardarson, 2004, Hardarson et al., 1993, Peoples et al., 2009). We conducted our evaluation in a Mollisol with adequate soil organic matter content that can limit the expression of SNF activity. Nonetheless, in the irrigated treatment, %Ndfa-G presented a mean value of 48% for two seasons or a mean value of 40 kg ha^−1^ of N fixed. Several genotypes presented %Ndfa-G values superior to 50%, representing more than 50 kg ha^−1^ of N fixed under irrigated conditions. The efficiency of major food crops in the recovery of applied N is often not more than 30% (Subbarao et al., 2013); so 50 kg N fixed through SNF would be comparable to about 150 kg N applied as chemical fertilizer. Line RCB 593 suffered less inhibition of SNF ability under drought stress when compared to irrigated conditions; moreover, and it presented the highest SNF ability under drought stress. This line could be a potential parent to improve SNF capacity under drought stress in bush beans. Other lines that were resistant to drought and showed relatively higher SNF ability included BFS 29, BFS 32, SER 48, SEA 15 and NCB 226. The drought- adapted line NCB 226 has previously been found to be superior in its SNF ability under drought stress using the methodology of acetylene reduction activity (ARA) determined with a flow-through system (Devi et al., 2013). Our results also indicated that the drought resistant check BAT 477 showed moderately high %Ndfa under drought stress. This line has previously been identified with good SNF ability both under optimal (Kipe-Nolt and Giller, 1993) and drought stress conditions (Castellanos et al., 1996). As expected, most drought sensitive lines were poor in their SNF ability, for example, DOR 390, a commercial variety used widely in at least three countries in Latin America.

This study also found that all the best N fixers under drought were drought resistant lines, confirming the tendency that was observed in previous studies with common bean (Beebe et al., 2014, Devi et al., 2013). A positive and significant correlation was observed between grain yield and TNdfa (kg ha^−1^) in grain under both irrigated and drought conditions, indicating that the genotypes with more N accumulation from fixation presented higher grain yield under both irrigated and drought conditions. The lines RCB 593, SEA 15, BFS 29, SCR 16, NCB 280 and NCB 226 accumulated more N from symbiotic fixation, and used it for greater grain production both under drought and irrigated conditions. Several lines that performed well under drought stress, such as SMC 141, RCB 593, BFS 32, SEN 56 and NCB 226, combined greater SNF ability with more efficient use of N to produce grain. Despite the fact that SNF ability can be drastically affected by drought stress, the more drought resistant Middle American lines overcome this limitation by using the acquired N more efficiently through greater remobilization of both C and N to grain. A higher use efficiency of the acquired N can be very relevant for crop yield in environments dominated by strong droughts where the viability of *Rhizobium* and in consequence SNF is severely inhibited.

## 5 Conclusions

Correlations between %Ndfa using shoot tissue (%Ndfa-SH) and %Ndfa using grain tissue (%Ndfa-G) indicated that the values of %Ndfa-G can be used to quantify phenotypic differences in symbiotic nitrogen fixation (SNF) of common bean under either irrigated or drought stress conditions. Estimates of %Ndfa-G are easier to implement in a breeding program due both to less labor costs and the feasibility to determine this parameter at harvest time. Using %Ndfa-G values, we observed significant phenotypic differences in SNF ability in common bean under drought stress. We identified four bean lines RCB 593, SEA 15, NCB 226 and BFS 29 that were not only drought resistant but also were superior in their SNF ability and these lines could serve as parents in breeding programs. Our results also indicate that the drought response of the SNF ability of tepary bean (*P. acutifolius*) and common bean are different, possibly due to differences in internal regulation mechanisms of SNF.

## Figures and Tables

**Table 1:** Correlation coefficients (r) between% nitrogen derived from the atmosphere estimated using shoot tissue (%Ndfa-SH), % nitrogen derived from the atmosphere estimated using grain tissue (%Ndfa-G), total nitrogen derived from the atmosphere in kg ha^−1^ using grain tissue (TNdfa-G), total nitrogen derived from the soil in kg ha^−1^ using grain tissue (TNdfs-G), nitrogen use efficiency in kg of grain produced kg^−1^ of N uptake in the shoot (NUE), shoot biomass in kg ha^−1^ (SB) and grain yield in kg ha^−1^ (GY) of 36 bean genotypes of grown under irrigated and drought conditions in a Mollisol at CIAT-Palmira, Colombia. Values reported are from analysis of data collected from two seasons of evaluation (2013 and 2014).

*Irrigated*							
Trait	%Ndfa-SH	%Ndfa-G	TNdfa-G	TNdfs-G	NUE	SB	GY
%Ndfa-SH	1						
%Ndfa-G	0.83***	1					
TNdfa-G	0.65***	0.82***	1				
TNdfs-G	−0.53***	−0.69***	−0.29***	1			
NUE	0.06	0.07	0.32***	0.15*	1		
SB	0.07	0.05	0.20**	0.32***	−0.45***	1	
GY	0.16*	0.16*	0.61***	0.50***	0.51***	0.39***	1

**Drought**							
Trait	%Ndfa-SH	%Ndfa-G	TNdfa-G	TNdfs-G	NUE	SB	GY
%Ndfa-SH	1						
%Ndfa-G	0.71***	1					
TNdfa-G	0.56***	0.83***	1				
TNdfs-G	−0.22**	−0.37***	0.09	1			
NUE	0.20**	0.19**	0.45***	0.48***	1		
SB	−0.13	−0.16*	0.17*	0.57***	−0.12	1	
GY	0.05	0.05	0.51***	0.86***	0.61***	0.59***	1

*, **, *** Significant at the 0.05, 0.01 and 0.001 probability levels, respectively.

**Table 2:** Phenotypic differences in% nitrogen derived from the atmosphere estimated using shoot tissue (%Ndfa-SH), % nitrogen derived from the atmosphere estimated using grain tissue (%Ndfa-G), shoot ^15^N natural abundance and grain ^15^N natural abundance of 36 genotypes of common bean grown under irrigated and drought conditions in 2012 and 2013 at Palmira, Colombia.

Genotype	%Ndfa-Shoot				%Ndfa-Grain				^15^N natural abundance in shoot (‰)				^15^N natural abundance in grain (‰)			
	Irrigated		Drought		Irrigated		Drought		Irrigated		Drought		Irrigated		Drought	
	2012	2013	2012	2013	2012	2013	2012	2013	2012	2013	2012	2013	2012	2013	2012	2013
ALB 6	57	50	22	25	53	44	17	29	0.6	2.5	5.9	6.3	1.4	2.5	6.3	5.1
ALB 60	56	39	27	26	58	38	27	20	0.7	3.8	5.4	6.1	0.9	3.0	5.3	6.2
ALB 74	55	36	28	16	60	42	18	18	0.8	4.1	5.3	7.3	0.7	2.7	6.2	6.3
ALB 88	62	31	9	23	66	34	7	18	0.2	4.7	7.5	6.5	0.3	3.5	7.4	6.3
ALB 213	49	47	18	29	62	30	12	23	1.3	2.8	6.4	5.7	0.6	3.8	6.9	5.8
BAT 477	58	49	17	32	60	44	11	30	0.2	2.5	6.4	5.1	0.5	2.4	6.9	4.8
BAT 477_NN	0	0	0	0	0	0	0	0	5.6	8.3	8.7	9.5	5.8	6.8	8.6	8.3
BFS 10	58	34	18	28	65	40	18	22	0.5	4.4	6.4	5.8	0.4	2.9	6.1	5.8
BFS 29	59	42	24	25	70	40	25	29	0.4	3.5	5.8	6.3	0.0	2.9	5.5	5.2
BFS 32	61	45	26	23	58	39	34	25	0.2	3.1	5.5	6.5	1.0	2.9	4.5	5.6
BFS 67	61	41	29	26	62	41	12	27	0.2	3.6	5.2	6.2	0.7	2.8	6.9	5.3
DOR 390	58	38	25	23	58	42	20	19	0.6	3.9	5.6	6.5	0.9	2.7	6.0	6.2
G 40001	52	28	11	7	54	18	1	12	1.0	5.1	7.3	8.5	1.3	4.8	8.0	7.0
INB 827	60	36	25	30	68	39	18	27	0.3	4.1	5.6	5.6	0.2	3.0	6.1	5.3
INB 841	55	37	19	25	61	24	16	27	0.8	4.1	6.3	6.2	0.7	4.3	6.4	5.3
MIB 778	56	50	15	14	62	45	4	16	0.7	2.6	6.9	7.6	0.6	2.4	7.7	6.5
NCB 226	62	52	15	37	69	44	14	32	0.2	2.4	6.8	4.7	0.1	2.6	6.6	4.8
NCB 280	45	24	16	26	60	30	17	26	1.7	5.5	6.7	6.1	0.7	3.8	6.4	5.4
Pérola	57	45	21	38	59	45	23	24	0.3	2.9	6.1	4.3	0.6	2.3	5.6	5.6
RCB 273	55	48	13	25	62	36	17	23	0.8	2.9	7.0	6.3	0.6	3.3	6.3	5.7
RCB 593	45	38	35	33	55	37	31	36	1.6	3.8	4.5	5.2	1.2	3.1	4.9	4.4
SCR 2	56	23	13	34	58	22	9	25	0.6	5.6	6.9	5.1	1.0	4.5	7.1	5.6
SCR 9	63	32	23	21	65	39	19	26	0.1	4.6	5.8	6.7	0.3	3.0	6.2	5.5
SCR 16	58	35	20	25	59	37	26	27	0.5	4.3	6.2	6.2	0.9	3.2	5.4	5.4
SEA 15	55	38	34	35	60	35	19	22	0.5	3.7	4.4	4.8	0.5	3.2	6.0	5.9
SEN 56	63	41	17	28	67	30	21	20	0.1	3.6	6.6	5.8	0.3	3.8	5.9	6.1
SER 16	58	39	19	24	59	33	13	21	0.5	3.8	6.3	6.4	0.9	3.5	6.7	6.0
SER 48	66	42	27	27	61	44	23	32	−0.2	3.5	5.4	6.0	0.7	2.6	5.6	4.9
SER 78	59	31	33	25	63	31	17	23	0.4	4.6	4.7	6.2	0.6	3.6	6.3	5.8
SER 118	59	37	20	18	65	41	26	13	0.4	4.0	6.2	7.1	0.4	2.8	5.4	6.9
SER 119	66	52	26	30	66	45	16	22	−0.1	2.3	5.6	5.6	0.3	2.5	6.4	6.0
SER 125	62	40	20	30	64	39	27	28	0.1	3.7	6.2	5.6	0.5	3.0	5.2	5.3
SMC 43	56	55	21	25	64	47	13	23	0.7	2.1	6.1	6.2	0.5	2.2	6.7	5.7
SMC 141	57	51	31	20	60	53	22	28	0.6	2.4	5.0	6.8	0.8	1.7	5.7	5.3
SXB 412	47	41	17	26	51	47	18	24	1.2	3.4	6.5	5.9	1.3	2.1	6.1	5.5
Tio Canela 75	53	37	42	23	57	45	34	17	0.9	4.0	3.6	6.4	1.1	2.5	4.5	6.4

Mean	**56**	**39**	**22**	**25**	**59**	**37**	**18**	**23**	**0.7**	**3.8**	**6.0**	**6.2**	**0.8**	**3.1**	**6.2**	**5.8**
Sig. diff.	*	*	*	*	*	*	*	*	*	*	*	*	*	*	*	*

*Significant difference at 0.05 level as estimated from the MIXED procedure.
